# Supplementary material for: A lipid metabolism-related gene signature reveals dynamic immune infiltration of the colorectal adenoma-carcinoma sequence
Source: Lipids Health Dis. 2023 Jul 4;22:92. doi: 10.1186/s12944-023-01866-4 (PMC10318759; doi:10.1186/s12944-023-01866-4)
Supplement: Supplementary file 3 — Supplementary Material 3 [file 12944_2023_1866_MOESM3_ESM.pdf]

PAPER NAME

A lipid metabolism-related gene signatur e reveals dynamic immune infiltration of the colorectal aden

WORD COUNT

2905 Words

CHARACTER COUNT

17310 Characters

PAGE COUNT

11 Pages

FILE SIZE

113.0KB

SUBMISSION DATE

May 31, 2023 11:41 PM GMT+8

REPORT DATE

May 31, 2023 11:42 PM GMT+8

7% Overall Similarity

The combined total of all matches, including overlapping sources, for each database.

- 6% Internet database
- 5% Publications database
- Crossref database
- Crossref Posted Content database
- 0% Submitted Works database

Excluded from Similarity Report

- Bibliographic material

A lipid metabolism-related gene signature reveals dynamic immune infiltration of the colorectal adenoma-carcinoma sequence

Abstract

Background: Lipid metabolism-related genes (LMRGs) have been reported to be correlated with the immune infiltration of colorectal cancer (CRC). This study aimed to investigate the immune infiltration characteristics along the colorectal adenoma-carcinoma sequence (ACS) based on LMRGs.

Methods: Gene expression data of colorectal adenoma and carcinoma samples were obtained from the public databases. The "limma" package was applied to determine the differentially expressed LMRGs. Unsupervised consensus clustering was used to cluster colorectal samples. The features of the tumor microenvironment were analyzed by the "ESTIMATE", "GSVA", and "TIDE" algorithms.

Results: The expression of 149 differentially expressed LMRGs was defined as the LMRG signature. Based on this signature, the adenoma and carcinoma samples were divided into three clusters. Unexpectedly, these sequential clusters showed a directional relationship and collectively constituted the progressive course of colorectal ACS. Interestingly, the LMRG signature revealed that adenoma progression was accompanied by a progressive loss of immune infiltration and a stepwise establishment of a cold microenvironment, but carcinoma progression was characterized by a progressive gain of immune infiltration and a gradual establishment of a hot microenvironment.

Conclusions: The LMRG signature reveals dynamic immune infiltration along the colorectal ACS, which substantially changes the understanding of the tumor microenvironment of CRC carcinogenesis and provides novel insight into the role of lipid metabolism in this process.

Keywords: colorectal cancer, adenoma-carcinoma sequence, lipid metabolism, immune infiltration,

tumor microenvironment.

Introduction

In 2020, global cancer statistics reported 1.8 million new cases and 880,800 deaths due to colorectal cancer (CRC) [1]. Most cases of CRC are sporadic, accounting for over 80% of all diagnoses [2]. There is an established model of sporadic CRC known as the adenoma-carcinoma sequence (ACS), which describes the progressive process from normal mucosa to adenoma to carcinoma and is responsible for 85–90% of sporadic CRC cases [3].

CRC risk is predominantly driven by environmental factors, particularly a high-fat diet. Dysregulation in lipid metabolism has been found to be the most prominent metabolic alteration in cancer. Abnormal lipid metabolism can promote tumor growth and progression by modulating ferroptosis-induced cell death, promoting metastasis, and interacting with the tumor microenvironment [4, 5]. Moreover, activating oncogenes drive abnormal lipid metabolism to provide cancer cells with energy, lipid components, and signaling molecules that are needed for proliferation, invasion, and metastasis [4]. In particular, a high-fat diet is a highly relevant risk factor in CRC and is associated with nearly 80% of CRC cases [6]. Regardless of the formation and cancerous transformation of adenomas or the progression and metastasis of CRC, a high-fat diet is an important promoting factor [7, 8]. Therefore, understanding the role of abnormal lipid metabolism in CRC carcinogenesis is of great interest.

Tumor heterogeneity is a significant characteristic of cancers and a great challenge in the oncology field. Tumor heterogeneity shapes the tumor microenvironment and plays decisive roles in tumor initiation, progression, and drug resistance [9]. Several studies have provided novel insight into lipid metabolic heterogeneity in CRC based on lipid metabolism-related genes (LMRGs) and

reported that the LMRGs were associated with immune infiltration and were useful for predicting the prognosis of CRC patients [10-12]. However, no study has focused on the role of LMRGs in the complete course of the colorectal ACS. In particular, the relationship between LMRGs and immune infiltration along the colorectal ACS is unclear. This study aimed to investigate the immune infiltration characteristics along the ACS based on LMRGs.

Methods

Data collection and preparation

The gene expression datasets GSE41657 (12 normal, 51 adenoma, and 25 carcinoma samples), GSE37364 (38 normal, 29 adenoma, and 27 carcinoma samples), and GSE117606 (65 normal, 69 adenoma, and 74 carcinoma samples) were obtained from the Gene Expression Omnibus (GEO) repository, and RNA sequencing data of patients with colon adenocarcinoma (COAD, n=429) were downloaded from The Cancer Genome Atlas (TCGA) database.

A total of 776 human LMRGs were obtained from 7 gene sets in the Molecular Signature Database (http://gsea-msigdb.org) (**Table 1**) [13]. As a supplement, 833 LMRGs were preliminarily obtained by taking the union of the above 776 LMRGs and the LMRGs reported by Sun X et al. [14]. However, only 711 genes were used for differential expression analyses because not all the LMRGs could be detected during sequencing (**Sheet 1**).

Differentially expressed LMRG analyses and pathway enrichment analyses

The GSE41657 and GSE37364 datasets were adopted to screen the differentially expressed LMRGs among normal mucosa, colorectal adenoma, and colorectal carcinoma. Each dataset was analyzed independently, and the differentially expressed LMRGs were taken from their intersection. Principal component analysis was used to identify potential outliers in each dataset. The "limma"

package [15] was used to determine the differentially expressed LMRGs with adjusted *P* values less than 0.05 and absolute value of fold change more than 1.5 as the threshold. Kyoto Encyclopedia of Genes and Genomes (KEGG) and Gene Ontology (GO) analyses were used to elucidate the function of differentially expressed LMRGs by the "clusterProfiler" package [16].

Unsupervised consensus clustering analyses

Unsupervised consensus clustering is a popular method to identify robust clusters and has been extensively utilized in analyzing omics data [17-19]. The "ConsensusClusterPlus" package [20] was used to apply unsupervised consensus clustering to facilitate the classification of colorectal tumors based on the transcriptome of differentially expressed LMRGs, and repeated subsampling was set 500 times to ensure reliability. For the convenience of analysis, the colorectal adenoma and carcinoma samples were divided into three clusters based on the consensus clustering algorithm (adenoma: A1, A2, and A3; carcinoma: C1, C2, and C3). The t-distributed stochastic neighbor embedding (t-SNE) was applied to determine the difference in each cluster by the "Rtsne" package [21] based on the expression of differentially expressed LMRGs.

Evaluation of the tumor microenvironment

The stromal, immune, and ESTIMATE scores were calculated for each sample using the ESTIMATE algorithm [22]. The stromal score and immune score positively correlate with the matrix and immune in the tumor microenvironment, and the ESTIMATE score positively correlates with tumor purity. Gene set variation analyses were used to quantify the immune cells according to the 28 immune cell gene sets retrieved from a previous study [23] by the "GSVA" package. The Tumor Immune Dysfunction and Exclusion (TIDE) algorithm was used to calculate the antitumor immunotherapeutic response [24], which includes the dysfunction score of T lymphocytes,

exclusion score of T lymphocytes, and dysfunction and exclusion of T lymphocytes (TIDE score). A higher dysfunction score, exclusion score, and TIDE score indicate a higher likelihood of antitumor immune dysfunction or escape.

Statistical analysis

Statistical analyses were performed with R software (version 4.2.2). Kaplan–Meier (K–M) survival analyses were performed with the "survival" and "survminer" packages. The log-rank test was used to estimate the differences among K–M survival curves. Sankey plots were generated with the "ggalluvial" package [25]. Differences of continuous variables were calculated using a t test or analysis of covariance. All statistical tests were two-sided, and a *P* value less than 0.05 was considered significant.

Results

Identification of differentially expressed LMRGs

The GSE41657 and GSE37364 datasets were used to identify the differentially expressed LMRGs. Six overlapping upregulated and 22 overlapping downregulated LMRGs in the colorectal adenoma compared with the normal mucosa (**Figure 1A**), 38 overlapping upregulated and 103 overlapping downregulated LMRGs in the colorectal carcinoma compared with the normal mucosa (**Figure 1B**), and 13 overlapping upregulated and 16 overlapping downregulated LMRGs in the colorectal carcinoma compared with the colorectal adenoma (**Figure 1C**), were identified. In total, 149 unique LMRGs were identified (**Sheet 1**), which reflected the serious lipid metabolism disorders in colorectal tumors. The enriched GO and KEGG analyses suggested that the 149 LMRGs constituted the lipid component of cells, performed the molecular function of lipid metabolism, participated in the biological process of fatty acid metabolism, and affected a wide range of lipid

metabolic pathways (**Figure 1D and E**).

The LMRG signature reveals a progressive loss of immune infiltration in colorectal adenoma

The expression of the 149 LMRGs was defined as an LMRG signature. Based on the LMRG signature, the colorectal adenoma samples from the GSE41657 dataset were divided into three clusters (A1, A2, and A3) (**Figure 2A**). Gene set variation analysis suggested that most of the 28 immune cells were reduced in the adenoma samples compared with the normal samples (**Figure 2B**). Type 1 T helper cells, myeloid-derived suppressor cells, effector memory CD8 T cells, plasmacytoid dendritic cells, CD56 dim natural killer cells, active dendritic cells, and activated CD8 T cells showed a sequential decreasing trend in the three clusters (**Figure 2C**). The ESTIMATE algorithm showed a stepwise decreasing trend in the stromal, immune, and ESTIMATE scores in the three sequential clusters (**Figure 2D-F**), which suggested gradually reduced immune infiltration and a gradually colder microenvironment in the three sequential clusters. In particular, adenoma with high-grade dysplasia accounted for 21.1% of the samples in cluster A1 but accounted for 90.9% of the samples in cluster A3 (**Figure 2G**), which indicated that the three sequential clusters represented the progression of colorectal adenoma. Therefore, the three sequential clusters determined by the LMRG signature revealed a progressive loss of immune infiltration and a stepwise establishment of a cold microenvironment in colorectal adenoma (**Figure 2H**).

The LMRG signature reveals a progressive gain of immune infiltration in colorectal adenocarcinoma

A total of 429 colon carcinoma from the TCGA-COAD cohort were grouped into three clusters (C1, C2, and C3) (**Figure 3A**). Substantially greater immune infiltration was found in cluster C3 (**Figure 3B**). The stromal, immune, and ESTIMATE scores showed gradual gains in these three

clusters (**Figure 3C-E**). In addition, gradually increased dysfunction scores, exclusion scores, and TIDE scores were observed in the three sequential clusters (**Figure 3F-H**). Therefore, the sequential clusters C1, C2, and C3 manifested a progressive gain of immune infiltration with a gradually hot microenvironment and a progressively increased risk of antitumor immune escape. Antitumor immunity can be divided into three types: the immune-desert phenotype, inflamed phenotype, and immune-excluded phenotype [26]. Cluster C1 represented the immune-desert phenotype with no immunotherapeutic response because of an absolute lack of immune cells. Cluster C2 represented the inflamed phenotype that was amenable to immunotherapy because of numerous activated and semiactivated immune cells in the tumor microenvironment. Cluster C3 represented the immune-excluded phenotype that had poor effectiveness in immunotherapy because of the inhibition of abundant stromal elements [26]. No obvious differences were observed between the three clusters and TNM stage (**Figure 3I**). However, cluster C1 exhibited the best prognosis, and cluster C3 showed the worst prognosis (**Figure 3J**), as suggested by the K–M survival curve (log-rank P=0.011). As such, the three sequential clusters reflected the progressive development of colorectal carcinoma. The above results suggested that the three sequential clusters determined by the LMRG signature were associated with increased immune infiltration, increased antitumor immune escape, and reduced overall survival in colorectal carcinoma (**Figure 3K**), which was opposite to the change in the tumor microenvironment in colorectal adenocarcinoma.

The LMRG signature reveals dynamic immune infiltration along the ACS

The GSE117606 dataset was used to further confirm the above results. As before, both adenoma samples and carcinoma samples were grouped into three clusters (**Figure 4A and B**). The relative positions of normal, adenoma, and carcinoma samples exactly represented the course from

normal mucosa to adenoma to carcinoma as suggested by the t-SNE analysis (**Figure 4C**). Unexpectedly, the sequential clusters of colorectal adenoma and carcinoma showed a directional relationship and collectively constituted the progressive course of colorectal ACS (**Figure 4D**). In addition, the stromal, immune, and ESTIMATE scores decreased from cluster A1 to their lowest points in cluster A3 and then gradually rose to their highest points in cluster C3 (**Figure 4E**). The infiltration of most immune cells in these clusters also maintained almost the same trend (**Figure 4F**). Furthermore, the expression of CD3G (T-cell marker), CD8A ($CD8^+$ T-cell marker), CD19 (B-cell marker), and PD-L1 (an immunotherapy target of CRC) maintained the same trend as immune infiltration among these clusters (**Figure S1 A-D**). Eight LMRGs (CEBPB, ADH1B, PI3KCG, GLIPR1, CD36, CAV1, G0S2, and PTGS2) were also correlated with the characteristic immune infiltration along the colorectal ACS (**Figure S1 E-L**). The above evidence strongly demonstrated the rationality of analyzing the colorectal ACS based on the LMRG signature, and the LMRG signature revealed that adenoma progression was accompanied by a progressive loss of immune infiltration and a stepwise establishment of a cold microenvironment, but carcinoma progression was characterized by a progressive gain of immune infiltration and a stepwise establishment of a hot microenvironment.

Discussion

In this study, the expression of 149 differentially expressed LMRGs was defined as the LMRG signature. Based on the LMRG signature, the adenoma and carcinoma samples were divided into three clusters. Unexpectedly, these sequential clusters showed a directional relationship and collectively constituted the progressive course of colorectal ACS. Interestingly, the LMRG signature revealed that adenoma progression was accompanied by a progressive loss of immune infiltration

and a stepwise establishment of a cold microenvironment, but carcinoma progression was characterized by a progressive gain of immune infiltration and a gradual establishment of a hot microenvironment (**Figure S2**).

The ACS is responsible for 85–90% of sporadic CRC cases [27, 28], but it is a very slow process that takes approximately 10–20 years from a normal state to adenoma to cancer diagnosis. During this long course, a high-fat diet is the predominant environmental factor. Although a high-fat diet has been reported to be associated with many cancers, a high-fat diet is a particularly relevant risk factor in CRC and is associated with nearly 80% of CRC cases [6]. Therefore, understanding the role of lipid metabolism in CRC carcinogenesis is highly important. In the current study, 149 differentially expressed LMRGs were identified in colorectal adenoma and carcinoma, which reflects a serous lipid metabolism disorder in colorectal tumors. The clusters determined by the LMRG signature show a directional relationship and collectively constitute the progressive course of colorectal ACS. Perhaps most interestingly, the LMRG signature revealed a dynamic immune infiltration characteristic along the colorectal ACS. The above results reveal the important role of lipid metabolism in the tumor microenvironment of CRC.

Fatty acid oxidation can promote the effects of PD-1 antibody in $CD8^+$ T cells and enhance the survival of $CD8^+$ tissue-resident memory T cells, but excessive fatty acid oxidation inhibits the antitumor ability of effector T cells and supports the immunosuppressive function of regulatory T cells in the tumor microenvironment [29]. Thus, lipid metabolic alterations are closely related to the functional status of immune cells. A few studies have reported that LMRGs are immune-related prognostic indicators in various cancers, and many LMRG signatures have been constructed to evaluate the tumor microenvironment and identify different immune clusters [10, 11, 30, 31]. Unlike

previous studies, the current study did not emphasize the role of specific LMRGs but instead constructed clusters of colorectal tumors based on the LMRG signature. Surprisingly, evidence suggested that these clusters showed a directional relationship and collectively constituted the progressive course of colorectal ACS. In addition, this study did not focus solely on the clusters of carcinoma samples but rather on the complete process of colorectal ACS. Consequently, the current study revealed dynamic immune infiltration along the colorectal ACS based on the LMRG signature.

A previous study reported that tumor infiltration based on immune cell counts decreased in adenomas with low-grade dysplasia, adenomas with high-grade dysplasia, and invasive adenocarcinomas according to a small number of adenoma and carcinoma samples [32]; these findings share some similarities with those of the current research. However, the current study found that as adenomas undergo cancerous transformation, carcinoma progression is characterized by a progressive gain of immune infiltration. In fact, several studies have revealed the immune subtypes in COAD [33-35], and the immune subtypes in these studies also share similarity with the current research. However, they did not find a connection between different clusters. Unfortunately, there is no relevant research to elucidate the molecular mechanism by which immune infiltration in colorectal adenomas and carcinomas changes in this way. The progressive loss of immune infiltration in colorectal adenoma suggests a morbid exhaustion of immune cells that likely results from persistent antigen stimulation and chronic inflammation, and the progressive gain of immune infiltration in CRC suggests a diseased activation of immune cells that may contribute to the secondary response of the body's antitumor immunity.

Study strength and limitations

This study reveals dynamic immune infiltration along the colorectal ACS, which substantially

changes the understanding of the tumor microenvironment of CRC. The major limitation of this study is that all gene expression data were obtained from public databases and lack experimental validation in vivo and in vitro. However, similar results were obtained from different algorithms and multiple cohorts, which improves the accuracy and stability of the conclusions. Nevertheless, further experiments are necessary.

Conclusions

In summary, the LMRG signature reveals that adenoma progression is accompanied by a progressive loss of immune infiltration and a stepwise establishment of a cold microenvironment, but carcinoma progression is characterized by a progressive gain of immune infiltration and a gradual establishment of a hot microenvironment. The progressive loss of immune infiltration in colorectal adenoma suggests a morbid exhaustion of immune cells that likely results from persistent antigen stimulation and chronic inflammation, and the progressive gain of immune infiltration in colorectal carcinoma suggests activation of immune cells that may contribute to the secondary response of the body's antitumor immunity. These findings substantially change the understanding of the tumor microenvironment of CRC and provide novel insight into the role of lipid metabolism in this process.

7% Overall Similarity

Top sources found in the following databases:

- 6% Internet database
- 5% Publications database
- Crossref database
- Crossref Posted Content database
- 0% Submitted Works database

TOP SOURCES

The sources with the highest number of matches within the submission. Overlapping sources will not be displayed.

#	Source	Type	Similarity
1	ncbi.nlm.nih.gov	Internet	1%
2	Richard A. Hubner, Kenneth R. Muir, Jo-Fen Liu, Richard F.A. Logan, M...	Crossref	<1%
3	frontiersin.org	Internet	<1%
4	mdpi.com	Internet	<1%
5	Xin-Yu Ke, Miaowen Zou, Chenqi Xu. "Lipid metabolism in tumor-infiltra...	Crossref	<1%
6	bmccancer.biomedcentral.com	Internet	<1%
7	journals.plos.org	Internet	<1%
8	Pengxiang Li, Xuefeng Lv, Lu Liu, Mengle Peng, Dongchun Qin. "The Ro...	Crossref	<1%

9	bsdwebstorage.blob.core.windows.net<br>Internet	<1%
10	biorxiv.org<br>Internet	<1%
11	nature.com<br>Internet	<1%
12	Weina Yu, Qingyang Lei, Li Yang, Guohui Qin, Shasha Liu, Dan Wang, Yu...<br>Crossref	<1%
13	Zhi Li, Hua Zhang, Xixi Wang, Qun Wang, Jiapeng Xue, Yun Shi, Minghu...<br>Crossref	<1%
14	link.springer.com<br>Internet	<1%
15	Bihui Pan, Yue Li, Zhangdi Xu, Yi Miao et al. "Identifying a novel ferropt...<br>Crossref	<1%
16	Weijie Yu, Zhoulin Miao, Julaiti Ainiwaer, Bingzhang Qiao, Kawuli Juma...<br>Crossref posted content	<1%
