# Supplementary material for: A lipid metabolism-related gene signature reveals dynamic immune infiltration of the colorectal adenoma-carcinoma sequence
Source: Lipids Health Dis. 2023 Jul 4;22:92. doi: 10.1186/s12944-023-01866-4 (PMC10318759; doi:10.1186/s12944-023-01866-4)
Supplement: Supplementary file 4 — Supplementary Material 4 [file 12944_2023_1866_MOESM4_ESM.docx]

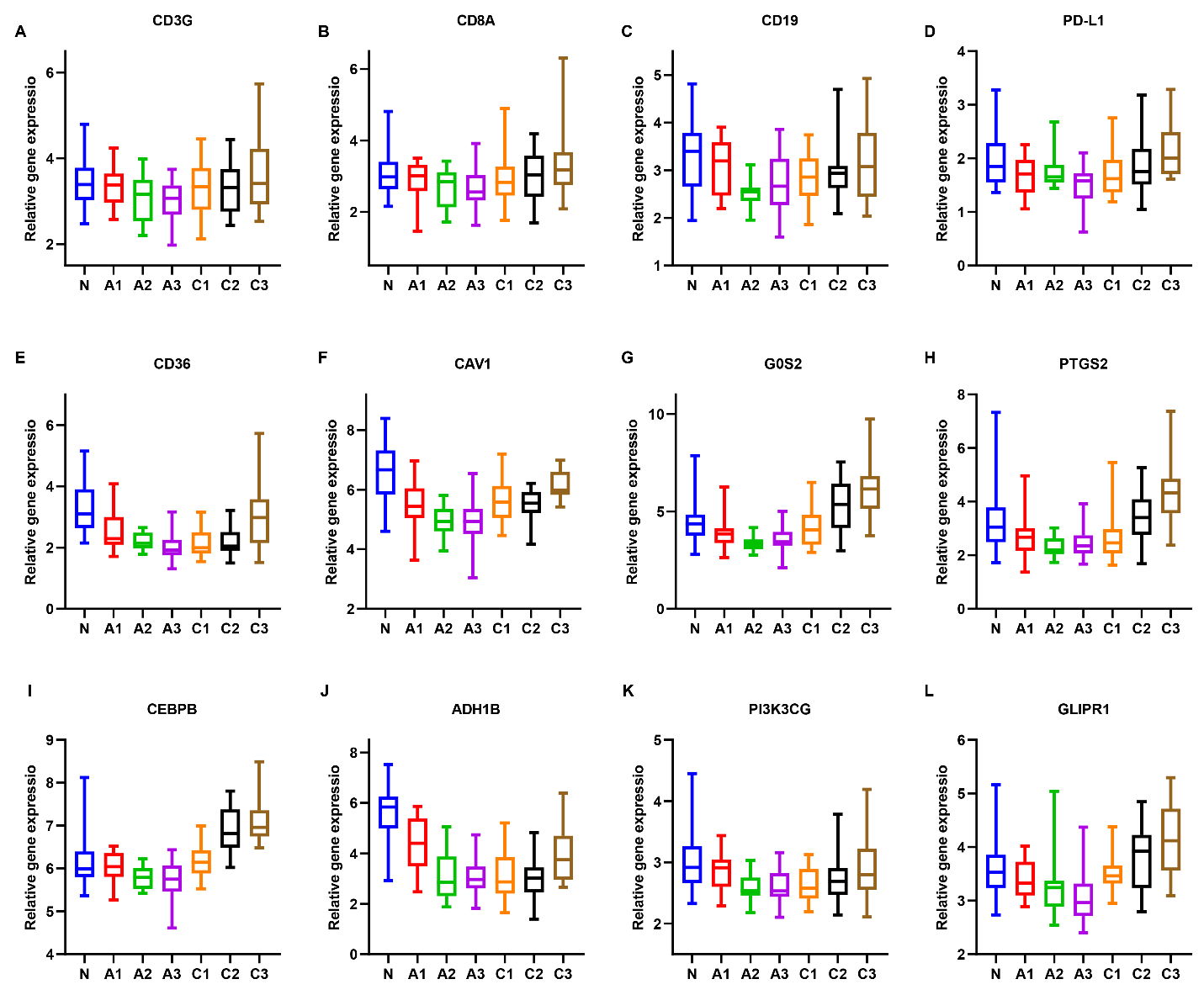


**Figure S1**. The expression of 12 genes in different subtypes in the GSE117606 database


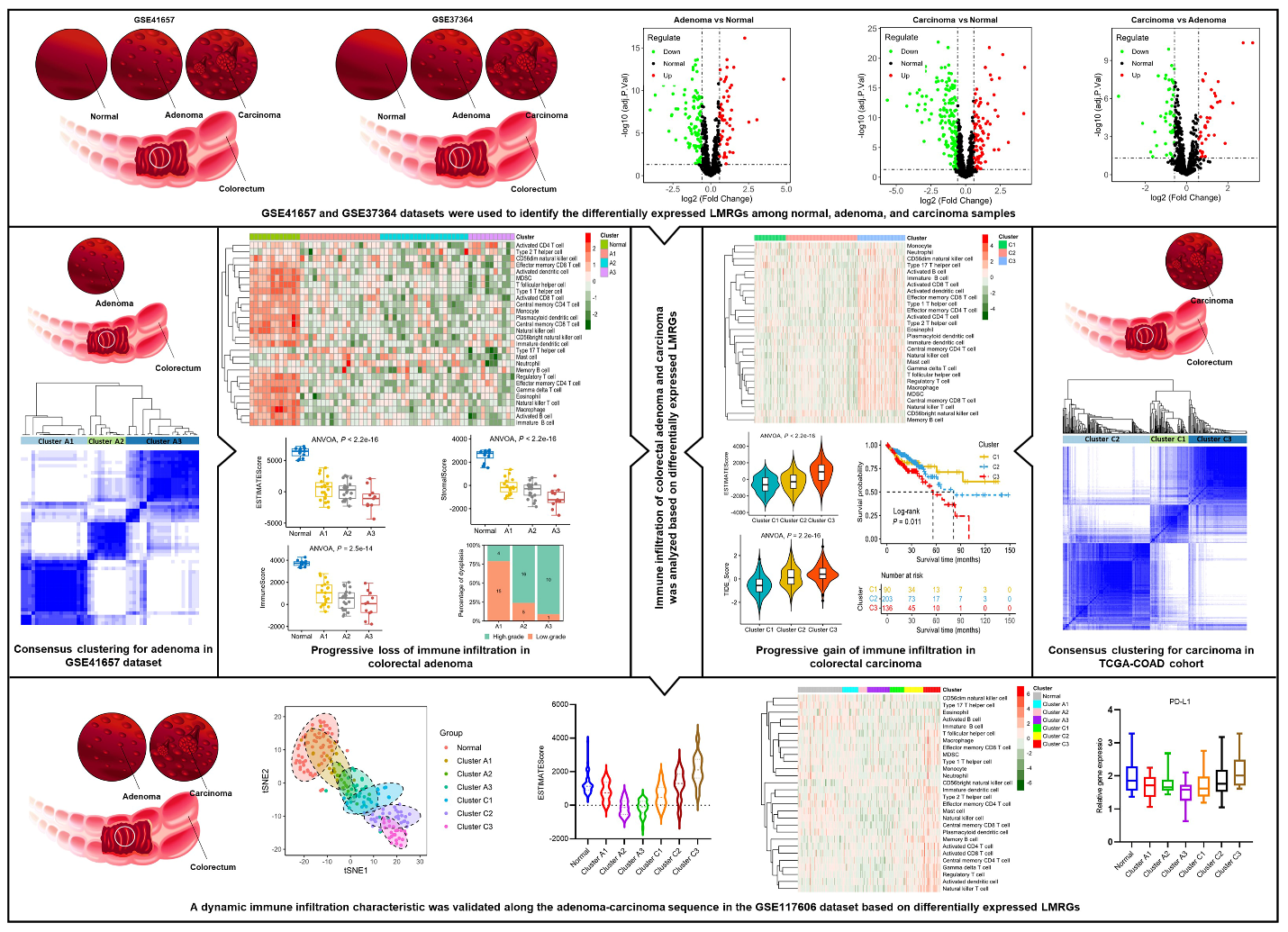


**Figure S2**. Graphical abstract. The LMRG signature that contained 149 differentially expressed LMRGs was obtained from the GSE41657 and GSE37364 databases. Based on the LMRG signature, the colorectal adenoma samples in the GSE41657 dataset and carcinoma samples in the TCGA-COAD cohort were divided into three clusters, and the immune infiltration characteristics in each cluster were analyzed. All the results were further validated in the GSE117606 dataset
